# SENSORY CHANGES IN MOUTH BREATHERS: SYSTEMATIC REVIEW BASED ON THE PRISMA METHOD

**DOI:** 10.1590/1984-0462/;2019;37;1;00012

**Published:** 2018-08-09

**Authors:** Ana Carollyne Dantas de Lima, Daniele Andrade da Cunha, Raquel Costa Albuquerque, Richelle Nogueira Alves Costa, Hilton Justino da Silva

**Affiliations:** a Universidade Federal da Paraíba, João Pessoa, PB, Brasil.; b Universidade Federal de Pernambuco, Recife, PE, Brasil.

**Keywords:** Mouth breathing, Sensation disorders, Somatosensory disorders, Child, Respiração bucal, Transtorno das sensações, Transtornos somatossensoriais, Criança

## Abstract

**Objective:**

To review, in the literature, information regarding changes in the sensory systems of mouth breathers.

**Data sources:**

The search was conducted in the following databases PubMed, BIREME, LILACS, Web of Science and Scopus. The search was independently carried out by two researchers, following the selection criteria. Original articles that approached mouth breathing and changes in sensory systems published in Portuguese, English and Spanish were published. Literature review of articles, dissertations, book chapters, case studies and editorials were excluded.

**Data synthesis:**

We found 719 articles. Among them, 663 were excluded by the title and 22 by the summary. Among the 34 analyzed manuscripts, 23 were repeated and 8 were excluded by reading the full text. Thus, 3 articles were selected for this review.

**Conclusions:**

Most studies presents the occurrence of changes in sensory systems in mouth breathing children. However, sensory reception is a matter of more concern. Besides, the evaluation of sensory systems was not standardized, which may have led to less precise results in the studied population.

## RESUMO

**Objetivo:**

Revisar, na literatura, estudos que abordem alterações nos sistemas sensoriais apresentadas por respiradores orais.

**Fonte de dados:**

A busca foi realizada nas bases de dados PubMed, BIREME, LILACS, *Web of Science* e *Scopus*. A busca foi realizada independentemente por dois pesquisadores, seguindo os critérios de seleção. Foram selecionados artigos originais que abordaram a respiração oral e as alterações nos sistemas sensoriais publicados nos idiomas português, inglês e espanhol. Os artigos de revisão da literatura, as dissertações, os capítulos de livros, os estudos de caso e os editoriais foram excluídos.

**Síntese dos dados:**

Foram encontrados 719 artigos, dos quais 663 foram excluídos pelo título e 22 pelo resumo. Trinta e quatro manuscritos foram analisados, dos quais 23 estavam repetidos e 8 foram excluídos pelo texto lido na íntegra. Assim, três artigos foram selecionados para esta revisão.

**Conclusões:**

A maioria dos estudos apresenta a ocorrência de alterações dos sistemas sensoriais em crianças respiradoras orais. Contudo, observa-se maior preocupação na avaliação da recepção sensorial. Além disso, a avaliação dos sistemas sensoriais foi realizada de forma não padronizada, o que pode ter acarretado resultados menos precisos na população estudada.

## INTRODUCTION

Breathing is a vital function normally carried out through the nasal airway, allowing that the inspired air, by passing through the nose, be purified, filtered, heated and humidified in the route to the lungs.^1^ ^,^ ^2^

Thus, this breathing method protects the upper airways and allows the proper development of the cranial-facial complex, being associated with normal mastication, swallowing, tongue and lip posture, besides providing the correct muscular action which stimulates the adequate facial growth and bone development.^2^ ^,^ ^3^ ^,^ ^4^ If there is any interruption in the air passage, obstructive or nonobstructive, the individual is led to breathe through the mouth.^1^ ^,^ ^5^ ^,^ ^6^

Usually, mouth breathing (MB) begins early, with causes associated to inflammation in the nasal cavity mucosa, pharyngeal and palatine tonsils, besides reduced or absent breastfeeding.^7^ ^,^ ^8^ The characteristics that are mostly present in MB in childhood are: frequent tiredness, daytime sleepiness, adynamia, nocturnal enuresis, reduced appetite, nutritional changes, learning deficit and damage in some sensory systems, with evidence of changes in the olfactory, and, consequently, gustatory system, besides the auditory system.^3^ ^,^ ^7^ ^,^ ^9^

All of this context presents the potential to affect sensory processing, a neurological function responsible for organizing and modulating the information received by the senses (palate, smell, vision, hearing, touch, movement, gravity and body position). This organization and modulation allow the human being to select the relevant information and respond adequately to the environment, which enables the realization of daily tasks.^10^ Sensory processing plays a major role in the executive functions of the individual, because, in order to carry out a motor action, previous sensory information is required^10^ ^,^ ^11^. The ideation, planning and execution of a motor action are functions of the central nervous system (CNS), called praxis, which depend on the full sensory modulation for its proper functioning. So, a flaw in sensory processing can bring sensory modulation disorders, as well as discrimination and praxis disorders.^11^

In Brazil, it is common to see mouth breathing in children at school age.^12^ At this age group, symptoms such as frequent tiredness, daytime sleepiness, reduced appetite, low oxygen in the brain, inability in auditory processing and concentration deficit, causing learning problems, are more common.^1^ ^,^ ^5^ ^,^ ^6^ At early ages, these changes can cause difficulties in speech and development of the child as a whole.^1^ ^,^ ^9^ ^,^ ^12^ Considering that this stage of life is very important for cognitive, motor and social formation of the individual, any change in the performance of its activities may lead to consequences in the formation of its occupational role. However, despite the usually early and continuous installation of respiratory damage, mouth breathers adapt to this situation and do not realize the impact generated on quality of life and deficits in functional performance.^1^ ^,^ ^6^ ^,^ ^9^

Therefore, considering breathing as a vital function to individuals and the damages caused by changes in the respiratory method, including the modulation of the information received by the environment, this study aimed at searching for evidence in the literature about the changes in sensory systems presented by mouth breathers. The objective of this study was to search, in the literature, systematically, for studies presenting the possible changes of sensory systems of children who breathe through their mouths.

## METHOD

For this review, the bibliographic research was based on the questions “Do mouth breathers present changes in sensory systems?”, and “How is the processing of sensory information in mouth breathers?”, which were based on the Population, Intervention, Comparison, Outcome (PICO) model, used in the Practice-Based-Evidence (PBE) and recommended for systematic reviews.^13^

The systematic reviews are based on clear questions, using systematized and explicit methods aiming at identifying, selecting and critically assessing relevant studies. In this sense, the choice was to use the PRISMA recommendation, a checklist with 27 items and 1 flowchart aiming at assisting authors to improve the quality of their systematic reviews and meta-analyses.^14^

Since the studies analyzed presented different characteristics (Tables 1, 2 and ">3), including heterogeneous sample, objectives, and methodological procedures, besides not reporting clinical trials, it was not possible to carry out their statistical analyses (meta-analysis). However, after data analysis, the survey enabled the establishment of considerations about the sensory changes presented by mouth breathers.

**Table 1:** Analyzed variables in the study by Correa et al.^16^

Author/ Year	Location	Sample	Assessed sensory system/evaluation	Study objectives	Results/ sensory changes
Correa et al., 2011.	Santa Maria, Rio Grande do Sul.	102 children (8 12 years old): Mouth breathing (n=52); Nasal breathing (n=50).	Auditory system/filtered speech test; standard frequency test; alternate disyllable dichotic test.	To highlight possible relationships between mouth breathing and central auditory system of the students.	Mouth breathing children present inferior performance in auditory processing than those with normal respiratory pattern; The evaluation of the auditory processing showed no association between the results of the different tests.

**Table 2:** Analyzed variables in the study by Roggia et al., 2010.^17^

Author/ Year	Location	Sample	Assessed sensory system/evaluation	Study objectives	Results/ sensory changes
Roggia et al., 2010.	Santa Maria, Rio Grande do Sul.	109 children (8 12 years old): Mouth breathing (n=51); Nasal breathing (n=58).	Visual, vestibular, somatosensory system/Dynamic posturography (sensory organization tests).	To compare posture and body balance among groups of students with and without mouth breathing, considering gender.	Mouth breathing students present postural changes in cephalic placement (female gender), and in lower limbs (male gender). Body balance of the mouth breathing students, in both genders, was more damaged in relation to those without mouth breathing, especially in the presence of sensory conflict.

**Table 3:** Variables analyzed in the study by Bianchini et al.,2009.^18^

Author/ Year	Location	Sample	Assessed sensory system/evaluation	Study objectives	Results/ sensory changes
Bianchini et al., 2009.	São Paulo, São Paulo.	97 mouth breathing children (5 12 years old).	Auditory system/ audiometry and tympanometry.	To verify the relationship between the etiology of mouth breathing and different types of auditory change.	Mouth breathers due to functional etiology had 100% of normal hearing, and, in the other etiologies, mild conductive hearing loss was prevalent, especially at the presence of palatine tonsil hypertrophy (adenoid), which causes more damage to the hearing system.

## DATA SOURCE

A search was conducted in the platforms PubMed and BIREME, and in the data bases MEDLINE, LILACS, Web of Science and Scopus, from January to February 2017. We used descriptors for the study (DECs and MeSH) - keywords to recover the subjects in the literature - and free terms (FT) - terms not found in DECs and MeSH, but relevant for the study. The crossings of these descriptors were carried out in English, Portuguese and Spanish as follows: Mouth Breathing (MeSH/DECs) AND Sensory Changes (FT) OR Sensation Disorder (MeSH/DECs) OR Somatosensory Disorder (MeSH/DECs) OR Smell Disordersn (MeSH/DECs) OR Smell (MeSH/DECs) OR Touch Perception (MeSH/DECs) OR Touch (MeSH/DECs) OR Labyrinth Vestibule (MeSH/DECs) OR Proprioception (MeSH/DECs) OR Visual changes (MeSH/DECs) OR Vision (MeSH/DECs) OR Taste Changes (MeSH/DECs) OR Taste (MeSH/DECs) OR Hearing Disorders (MeSH/DECs) OR Hearing (MeSH/DECs) OR Sensory Processing (FT).

As inclusion criteria, original articles approaching MB and changes in sensory systems were selected, focusing on the processing of the received information. The manuscripts were published in Portuguese, English and Spanish. The literature review articles, dissertations, book chapters, case studies and editorials were excluded, as well as those that did not present in the title, abstract or text the subject approached in this review. Manuscripts that did not specifically report the changes occurred in the sensory systems were also excluded.

The articles were selected based on the use of descriptors and FT defined, and the identification was carried out in three steps, as follows:

- Step 1: reading of the titles of the studies found, and exclusion of those that did not meet any of the inclusion criteria of this study;
- Step 2: reading of the abstracts of the studies selected in step 1, and exclusion of those that did not meet the inclusion criteria;
- Step 3: full reading of all studies left from the previous steps, and selection of those which met the inclusion criteria, using a protocol created for this purpose.

It is worth to mention that studies repeated in the different databases were only excluded after the full reading, preventing errors in the exclusions.

The articles that met all selection criteria and that enabled responses to the questions in this review were selected. The articles were assessed according to the critical review form for quantitative studies,^15^ whose objective is to provide recommendations and to assist the report of observational studies using a checklist.

The data of these articles were analyzed in detail through a protocol form created for this study. There, the following aspects were observed: author, year, location, population/sample, assessed sensory system, evaluation used, objective of the study, and main results.

The presentation of data considered the relevant points in each article using tables and figures, in order to observe and understand them during the presentation of results and discussion.

## RESULTS AND DISCUSSION

**Figure 1:**
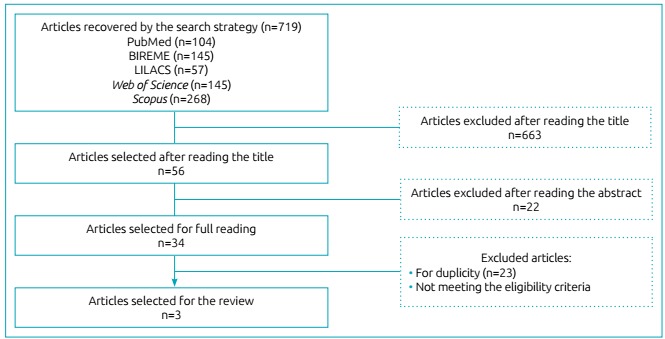
Flowchart of the number of articles found and selected after the application of the inclusion and exclusion criteria.

Seven-hundred and nineteen (719) articles were found after the search for the descriptors and FT. Of these, 104 were found in PubMed; 145 in BIREME; 57 in LILACS; 145 in Web of Science; and 268 in Scopus. According to the eligibility criteria, three articles were selected for this review, according to Figure 1.

The analyzed articles were published in the past eight years, since there were no studies found in the past six years. Besides, they were all carried out in Brazil, in two different regions (South and Southeast). These factors may be related to the use of the descriptors “mouth/oral breathing” for the collection of articles, and may possibly show the choice of Brazilian authors for defining MB no longer as a symptom of changes in the respiratory system, but as a set of clinical symptoms with varied etiology.^16^ ^,^ ^17^ ^,^ ^18^ This is shown by the fact that the three studies assessed define their study population as mouth breathers, based on a speech language diagnosis and observation of signs and symptoms. ^16^ ^,^ ^17^ ^,^ ^18^

The sample was another relevant aspect in the manuscripts, ranging between 97 and 109 individuals. ^16^ ^,^ ^17^ ^,^ ^18^ The population of the studies was mostly composed of children aged between 5 and 12 years. These findings can be understood because MB is common in the infant population, with studies presenting significant prevalence in this population.^9^ ^,^ ^19^ ^,^ ^20^ ^,^ ^21^ ^,^ ^22^ This finding can also be related with the concern of the authors regarding the development of these children, since some characteristics of the mouth breather syndrome are tiredness and frequent sleepiness, which lead to poor school performance and in common activities of childhood, such as games requiring more physical effort and attention.^9^ ^,^ ^19^ ^,^ ^20^ ^,^ ^21^

Another important factor is the relationship of causes of MB, such as early weaning, prolonged use of baby bottles and pacifiers, besides obstructive sleep disorders, very common in this population.^20^ ^,^ ^21^

Regarding the sensory systems presented, the studies approached the auditory (Tables 1 and ">3),^16^ ^,^ ^18^ visual, vestibular and somatosensory systems (Table 2)^17^ in a varied manner, observing the evaluation of all systems, except for the smell and taste systems.^12^ ^,^ ^14^ It is worth to mention that the touch system was not directly mentioned, however, we found a study that assessed the relationship of the somatosensory system of mouth breathers (Table 2),^17^ which is in accordance with some authors who consider the proprioceptive and touch systems as part of this system.^22^ ^,^ ^23^

Among the presented systems, the hearing one, despite not assessed in all articles, shows strong relationship with MB, due to the prevalence of chronic otitis as a consequence of the poor functioning of the auditory tube.^16^ ^,^ ^18^ ^,^ ^24^ Therefore, these changes may interfere in the capacity of speech sound perception, determining lack of attention and concentration, leading to developmental delay. ^18^ ^,^ ^24^

In spite of the close relationship between smell and taste, due to the excitement of taste receptors caused by the influence of smell, and the probable reduction of the latter because of MB,^25^ ^,^ ^26^ none of the studies selected for this review assessed these systems. The absence of studies with this subject in the review is possibly owed to the established eligibility criteria, since these data are usually found in manual and text books, which were not included in this study.

The article by Roggia et al.^17^ (Table 2) assesses the vestibular, visual and somatosensory systems in an integrated manner, presenting the influence of these three systems in the balance and posture of the oral breathing children. The authors relate the difficulties found with those of sensory conflicts, which, for Ayres, is called sensory integration.^10^ For this author, sensory integration refers to the organization of sensations for the use; that is, when the sensations flow in an organized and integrated manner, the brain can use them to form perceptions, behaviors and learning patterns. When the flow of sensations is disorganized, the individual may present what the author calls Sensory Processing Disorder, which leads to altered behaviors regarding response to the environment.^10^ ^,^ ^11^

Referring to the assessment instruments used in the studies, it was possible to observe the lack of standardization, that is, different types of evaluation were used for the same senses (Tables 1, 2 and ">3). ^16^ ^,^ ^17^ ^,^ ^18^

Even presenting the evaluation of the sensory systems, the objectives of the study are not addressed to evaluating the processing of sensory information or the relationship with the adaptive response to the environment (Tables 1, 2 and ">3). ^16^ ^,^ ^17^ ^,^ ^18^ The evaluation only addressed to the reception of sensations is remarkable, mostly disregarding the type of response that the altered central processing may bring to the individual’s behavior, and, consequently, for the performance of daily activities.^16^ ^,^ ^17^

Since sensory processing is in charge of organizing and modulating the information received by the senses, and plays an important executive role, the study of such processing and the adaptive responses given to the environment may justify many of the changes found in mouth breathers, such as lack of concentration, posture and gait changes, as well as differences in social relationship.^10^ ^,^ ^11^ ^,^ ^27^ Therefore, studies assessing the sensory processing of mouth breathers, using standardized instruments, are justified.

Despite the little relationship with the processing of sensory information, all results presented sensory changes in mouth breathing children, even when not associated with the consequences for the performance of the activities and the quality of life of these individuals (Tables 1, 2 and ">3). ^16^ ^,^ ^17^ ^,^ ^18^

The study by Correia et al*.* ^16^ (Table 1) presents inferior performance results in the auditory processing skills by mouth breathers; the analysis of Roggia et al.^17^ (Table 2) demonstrates damage in the body balance of this population, in comparison to nasal breathers. These data show the possible influence of breathing in sensory responses, and, consequently, changes in the performance of the infant population.

These findings encourage the need for further studies addressed to the relation of these sensory changes at a central level and the responses to the environment using the processing and perception of sensations. These studies could provide explanations for the behaviors found among mouth breathers, which influence the performance of their activities, and consequently, their quality of life.^10^ ^,^ ^11^ ^,^ ^15^ ^,^ ^27^

Therefore, the execution of studies that can deepen the knowledge about the relationship between sensory systems and the behavior of mouth breathing children is suggested, especially concerning the processing of sensory information, the integration of systems and the adaptive responses. Besides, it is important to prioritize the use of instruments that assess these points in a systematic and standardized manner, generating more accurate results.

## CONCLUSION

In this review, most studies showed the occurrence of changes in sensory systems among mouth breathing children. Despite these confirmations, there is greater concern in the evaluation of sensory reception, and not in the processing of information. Besides, most studies evaluated the sensory systems in a non-standardized manner, which may have led to less accurate results in the studied population.

The review in question showed the need to acquire more knowledge, in order to establish and standardize evaluation instruments for the sensory systems, since this study observed the lack of standardization of these instruments and a great variety in methodology, therefore reducing the reliability of the results.

Reaching this evaluation specificity will bring a more reliable diagnosis, and therapeutic planning based on scientific and reliable evidence, considering the relevance of the sensory systems for the performance of daily activities, and, consequently, for the quality of life.
